# Supplementary material for: Maize Genotypes Sensitive and Tolerant to Low Phosphorus Levels Exhibit Different Transcriptome Profiles under Talaromyces purpurogenus Symbiosis and Low-Phosphorous Stress
Source: Int J Mol Sci. 2023 Jul 26;24(15):11941. doi: 10.3390/ijms241511941 (PMC10418897; doi:10.3390/ijms241511941)
Supplement: Supplementary file 1 [file ijms-24-11941-s001.zip › Figure S1.pptx]

## Slide 1
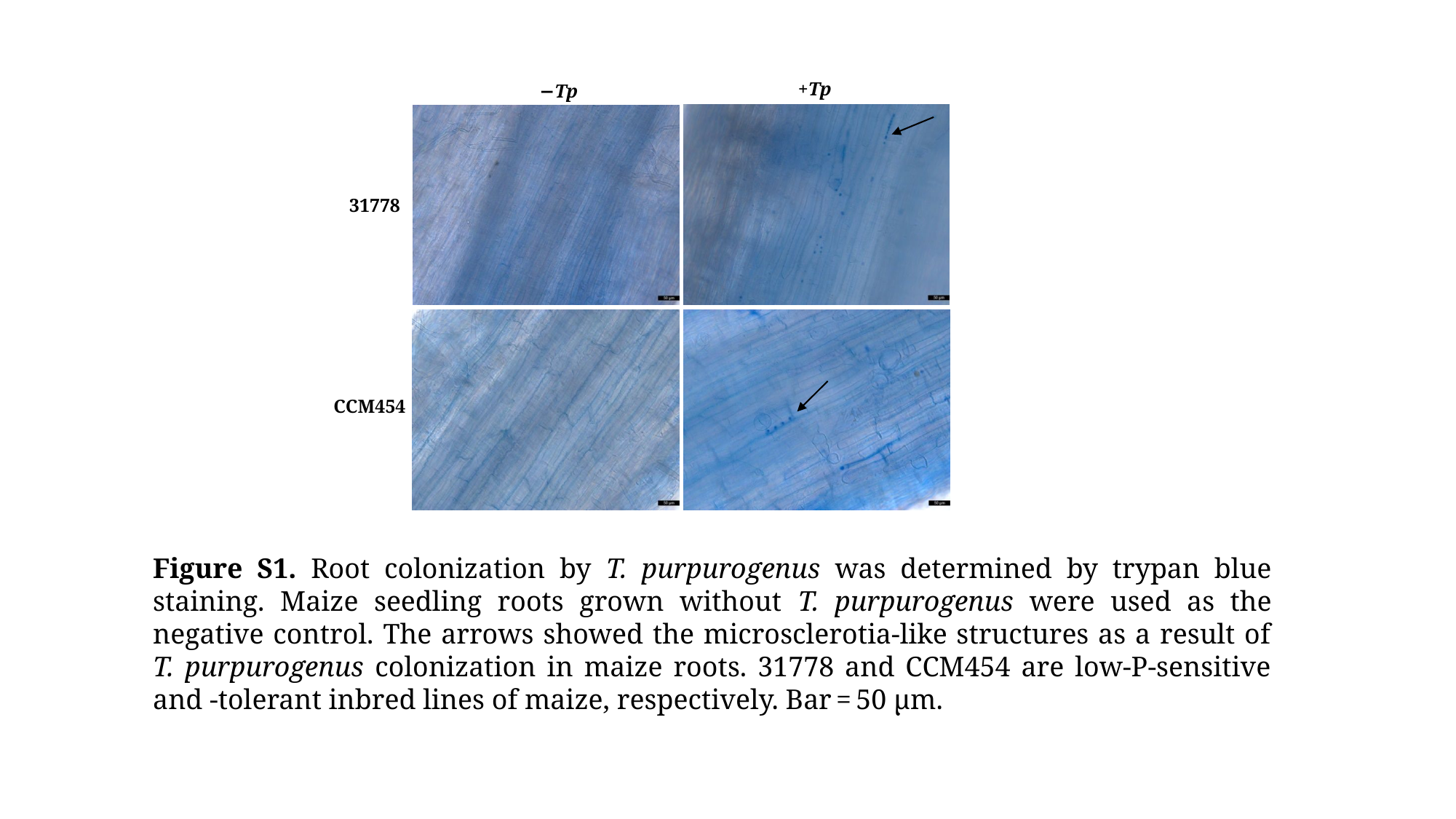

+Tp
−Tp
31778
CCM454
Figure S1. Root colonization by T. purpurogenus was determined by trypan blue staining. Maize seedling roots grown without T. purpurogenus were used as the negative control. The arrows showed the microsclerotia-like structures as a result of T. purpurogenus colonization in maize roots. 31778 and CCM454 are low-P-sensitive and -tolerant inbred lines of maize, respectively. Bar = 50 μm.
